# Epidemiology of Pediatric Head Trauma in Guilan

**DOI:** 10.5812/atr.7589

**Published:** 2012-10-14

**Authors:** Bruno Ramos Chrcanovic

**Affiliations:** 1 Department of Prosthodontics, Faculty of Odontology, Malmö University, Malmö, Sweden

**Keywords:** Brain Injury, Neurosurgery, Cognitive Aspect, Mental Health

*Dear Editor,*

Concerning the article “Epidemiology of Pediatric Head Trauma in Guilan” published in one of the previous issues of Archives of Trauma Research (1), I would like to make some considerations. Although the results of the Yousefzadeh Chabok et al.’s investigation are particularly interesting in terms of the lucid format used to present the data, it would be more interesting if the authors could have reported other injuries that are commonly associated with head traumas such as brains contusions, intracranial hematomas, fractures of the skull vault and of the skull base, external signs of trauma as scalp wounds or soft tissue hematomas, bleeding from the nose, ears, or throat, and uni- or bilateral involvement of one or more cranial nerves (2). Moreover, the authors might also have reported the influence of temporal factors in the incidence of head injuries in their study population, since the results of epidemiologic investigations vary depending on the demographics of the population studied. Factors such as geographic region, socioeconomic status, cultural differences, and temporal factors including time of the year and era, which could have impact on both the type and frequency of injuries in the population (2, 3). This sort of data is normally accessible from medical charts. In studies carried out in countries with four well-defined seasons (spring, summer, autumn, and winter), one might expect the summer months to yield a higher number of fractures (2-4). However, I believe that the four seasons are not well-defined throughout most of Iran (but I may be wrong). Another interesting point is that the distribution of fractures in the day of the week shows a predominant distribution of accidents causing facial and head injuries on the weekends (3-5). These are the days of great opportunity for outdoor and sports activities, little travels and recreations, because these are rest days. In some countries, these findings may also be related to increased alcohol and drug consumption during extended spare time activities and during weekend parties (3). However, this study was retrospective, and the nature of a retrospective study inherently results in flaws. These problems are manifested by the gaps in information and incomplete records. Furthermore, all data rely on the accuracy of the original examination and documentation. Items may have been excluded from the initial examination or not recorded in the medical chart (4, 5). Thus, the authors may have not found reported data of associated injuries or radiological documentation in a significant number (668 patients) as the medical charts of the study. As the authors stated in the article, “approximately 90% of the patients had no CT findings”. Unfortunately, unless you are conducting a prospective study, the researcher is subjected to this kind of limitation. An understanding of the patterns, etiologies, and consequences of trauma is essential so that the possible prevention of injuries and efficient allocation of health care resources can be prescribed. The descriptive studies are the first step to a better understanding of this health problem in a specific population level (4, 5). Thus, the authors are appreciated for completing this well-performed study concerning epidemiology of pediatric head trauma. This study is important because it helped to elucidate the causal pathway of such injuries in the mentioned Iranian province, and it will certainly help the health and social authorities to develop countermeasures to minimize the risk factors on this pathway, and thus reduce the incidence and severity of the injuries being targeted. 
